# Effect of Nano Nd_2_O_3_ on the Microstructure and High-Temperature Resistance of G@Ni Laser Alloying Coatings on Ti-6Al-4V Alloy

**DOI:** 10.3390/nano13061112

**Published:** 2023-03-20

**Authors:** Zifan Wang, Xiaoxi Meng, Zhihuan Zhao, Chuanzhong Chen, Huijun Yu

**Affiliations:** 1 Key Laboratory of High Efficiency and Clean Mechanical Manufacture, Ministry of Education, and National Demonstration Center for Experimental Mechanical Engineering Education, School of Mechanical Engineering, Shandong University, Jinan 250061, China; 2 Key Laboratory for Liquid-Solid Structural Evolution and Processing of Materials, Ministry of Education, and Shandong Engineering & Technology Research Center for Superhard Material, School of Materials Science and Engineering, Shandong University, Jinan 250061, China; 3 School of Mechanical and Electronic Engineering, Shandong Agricultural and Engineering University, Jinan 250100, China

**Keywords:** coating, high-temperature oxidation, laser alloying, nanoscaled neodymium sesquioxide, titanium alloy

## Abstract

Titanium and its alloys are widely used in high-end manufacturing fields. However, their low high-temperature oxidation resistance has limited their further application. Recently, laser alloying processing has attracted researchers to improve the surface properties of Ti, for which Ni coated graphite system is an excellent prospect due to its outstanding properties and metallurgical bonding between coating and substrate. In this paper, nanoscaled rare earth oxide Nd_2_O_3_ addition was added to Ni coated graphite laser alloying materials to research its influence on the microstructure and high-temperature oxidation resistance of the coating. The results proved that nano-Nd_2_O_3_ has an outstanding effect on refining coating microstructures, thus the high-temperature oxidation resistance was improved. Furthermore, with the addition of 1. 5 wt.% nano-Nd_2_O_3_, more NiO formed in the oxide film, which effectively strengthened the protective effect of the film. After 100 h of 800 °C oxidation, the oxidation weight gain per unit area of the normal coating was 14.571 mg/cm^2^, while that of the coating with nano-Nd_2_O_3_ addition was 6.244 mg/cm^2^, further proving that the addition of nano-Nd_2_O_3_ substantially improved the high-temperature oxidation properties of the coating.

## 1. Introduction

Due to their excellent properties, including high specific strength, strong corrosion resistance, lightweight, low thermal conductivity and excellent biocompatibility, titanium and its alloys are widely used in aerospace, automotive, chemical, medical and other high-end manufacturing fields [1,2,3]. However, the disadvantages of titanium alloys, such as poor high-temperature oxidation resistance have limited its further application in high-temperature component manufacturing fields. Titanium would easily react with oxygen in the air and then form loose and porous TiO_2_, which could exacerbate the oxidation of the alloy. Therefore, researchers have used various surface treatment techniques, including electroplating [4], chemical heat treatment [5,6,7], ion implantation [8,9], mechanical surface enhancement technology [10,11], and vapor deposition [12,13], to improve the properties of the surface to meet the application requirements.

Laser manufacturing technology has the advantages of high processing rate, superior machining precision, and extraordinary surface quality over traditional manufacturing methods [14]. With the development of laser manufacturing technology, laser surface modification, including laser shock strengthening [15], laser melting [16], laser deposition [17,18], and laser alloying [19,20], has attracted widespread attention in the field of Ti alloy surface technology. Among them, laser alloying technology is widely used for its advantages of fast heating speed, small heat-effected zone, tight bonding between coating and substrate, low deformation of the workpiece, and a high degree of automation [21]. Yamaguchi et al. [22] used laser alloying technology for the surface-hardening of AISI 304 austenitic stainless steels with light-transmitting resin as a source for the carbon element. After laser irradiation of a 304 stainless steel sheet laminated with titanium foil and transparent adhesive tape, a laser-alloyed zone of about 5 μm in thickness was obtained within fine titanium carbide particles formed by a reaction between titanium and pyrolytic carbon stemming from the resin, effectively improving the hardness of the substrate. Unfortunately, research on the laser alloying technology most focuses on the enhancement of hardness, wear resistance, corrosion resistance and other room temperature properties of the substrate, but its impact on high temperature properties, such as high-temperature oxidation resistance, high-temperature corrosion resistance and high-temperature fatigue properties, has been rarely studied.

In the process of laser surface modification, rare earth and its oxides have been widely considered as modification materials due to their special physical and chemical properties. Rare earth oxides have the properties of refining grains, improving the distribution of second-phase in the coating, and reducing the dilution rate, which can significantly improve the physical properties of the coating. Y_2_O_3_ [23], CeO_2_ [24], La_2_O_3_ [25] and Nd_2_O_3_ [26] are commonly used rare earth oxides that have the effect of grain refinement, microstructure improvement, and increasing the surface properties of the coating. Zhang et al. [27] successfully prepared Zr-based amorphous composite coatings with different amounts of Nd_2_O_3_ added to the AZ91D magnesium alloy with laser cladding technology. The results illustrated that appropriate Nd_2_O_3_ addition has the effect of refining the size of crystalline microstructure, purifying oxygen impurities and enhancing the amorphous content of the coatings, thus improving the hardness and corrosion resistance of the coating. Bu et al. [28] prepared Al-TiC and Al-TiC-Y_2_O_3_ coatings on the AZ63-Er alloy using laser cladding technology in order to improve the corrosion resistance of magnesium alloys for automobiles. With the addition of Y_2_O_3_, Al_3_Y and Al_4_MgY phases are newly formed, improving the hardness and corrosion resistance of the layer. 

Nanoscale rare earth oxides have strong acoustic, optic, electric, magnetic and thermodynamic characteristics because of the quantum size and surface effects [29], which are used in laser surface modification to refine the microstructures and reduce the tendency of cracking. Shi et al. [30] investigated the influence of nanoscaled rare earth La_2_O_3_ addition on the wear resistance of Ni60A/SiC laser cladding coating on the 60Mn steel substrate, and proved the improvement of the grain refinement and wear resistance of nano rare earth oxides. Zhang et al. [31] prepared laser cladding Ni-based alloy coatings on low carbon steel substrate with 1.5 wt.% nano-CeO_2_ and nano-Sm_2_O_3_ addition. Due to the thermodynamic characteristic of nanoscaled rare earth oxides, the morphology of dendrite in the coating has transformed from bulky into fine and compact, and the microhardness, wear resistance and corrosion resistance are greatly improved. However, almost all research focuses on the effect of rare earth oxides on hardness, wear resistance and corrosion resistance of the coating at room temperature. The influence of nano rare earth oxide addition on high-temperature oxidation behavior has rarely been involved. In this paper, the coatings on Ti-6Al-4V were fabricated by laser alloying using Ni-coated graphite materials with 1.5 wt.% nano Nd_2_O_3_ addition. The effects of Nd_2_O_3_ addition on the microstructure and high temperature oxidation resistance of coatings were investigated. This study aimed to further improve the high-temperature oxidation resistance of the laser alloying coatings on titanium alloy, and broaden the applications of titanium alloy in the field of high temperature resistance.

## 2. Materials and Methods

### 2.1. Materials

Ti-6Al-4V alloy was used as the substrate with a size of 10 mm × 10 mm × 10 mm. The substrates were abraded with 180# water sandpapers and cleaned with non-water ethanol using an ultrasonic cleaner. Ni-coated graphite (G@Ni) was used as the laser alloying powder, G@Ni contains graphite as the core and Ni as the coating with a chemical composition of 75% wt.% Ni + 25% wt.% graphite and particle size ranging 25–45 μm. The powder was mixed with a binder (Na_2_SiO_3_:H_2_O = 1:3), and then paved onto the Ti-6Al-4V samples. The thickness of the pre-paved coating is controlled to 1 mm. A certain amount of nano-Nd_2_O_3_ (1.5 wt.%) was added to the powder to compare with normal G@Ni laser alloying coating [32] and to explore its influence on the microstructure and high-temperature oxidation properties of the coating.

### 2.2. Laser Alloying and High-Temperature Oxidation Experiment

Laser alloying technology uses a high energy laser to scan and heat the pre-paved modification materials and the surface of the substrate. During the laser alloying process, the pre-paved materials melt and solidify rapidly, while reacting with the substrate sufficiently to form ceramic reinforcement-phases and intermetallic compounds. The obtained coatings can thus improve the surface properties, including the hardness, wear resistance, and high-temperature properties of the substrate. 

In this study, an American IPG Photonics YLS-4000 fiber laser with a wavelength of 1.064 μm and a light spot diameter of 4.0 mm was used for the laser alloying process. Argon was used to protect the molten pool from oxidation, with a gas flow of 10–15 L/min. The laser process parameters were determined according to early experiments: a laser power of 1400 W and a scanning speed of 15 mm/s. After laser alloying, the samples were cut along a cross-section in the vertical scanning direction, and were then polished by 180#, 400#, 600#, 800#, and 1000# water sandpapers in sequene until smooth and flat surfaces were obtained. The samples were polished with a Cr_2_O_3_ suspension and etched for 10–20 s in a mixed solution of HF and HNO_3_ (1:3, vol.%).

Before the high-temperature oxidation experiment, the surface of the Ti-6Al-4V and laser alloying samples were ground roughly. The samples were cleaned with absolute alcohol and dried for 1 h. The high-temperature oxidation test was conducted using a chamber electric furnace (CDRG SX2-12-12, Beijing, China) at 800 °C for 100 h. The oxidized samples were cooled and weighed in static air. The resistance of oxidation was judged by oxidation mass grain (ΔM).

### 2.3. Materials Characterization

**Figure 1:**
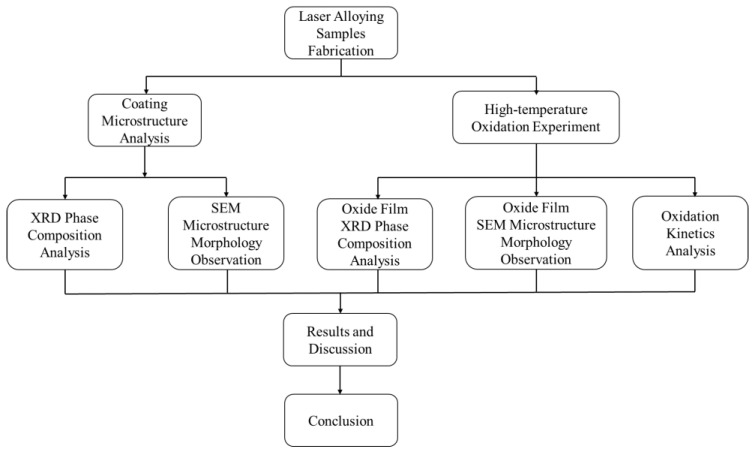
The experiment flow chart of this study.

The phase composition of the coating and the oxide film was analyzed using a Japanese Rigaku Ultima IV X-ray diffractometer, with a voltage of 40 kV, a current of 40 mA, a scanning range of 10°–90°, and a scanning speed of 2°/min. Morphologies of the coating, the surface and cross-section morphology of oxide film were observed using a Japanese Hitachi JSM-7800F scanning electron microscope, while the components of the oxide film were analyzed using the attached XMax-80 energy dispersive spectrometer. The area method was used to analyze typical microstructure morphology, i.e., typical microstructures were selected and their proportion was calculated using graphical processing software. The oxidation weight gain was tested with a German Sartorius QUINTIX-224 electronic balance, with a measuring range of 0.1 mg. The experiment flow chart of this study is shown in Figure 1.

## 3. Results and Discussion

### 3.1. Phase Compositions and Microstructure Morphology

The macroscopic surfaces of the coatings are continuous and homogeneous, with no obvious cracks or pores. This indicates an excellent surface macroscopic quality of the coatings for the selected material system and the laser alloying process parameters used.

**Figure 2:**
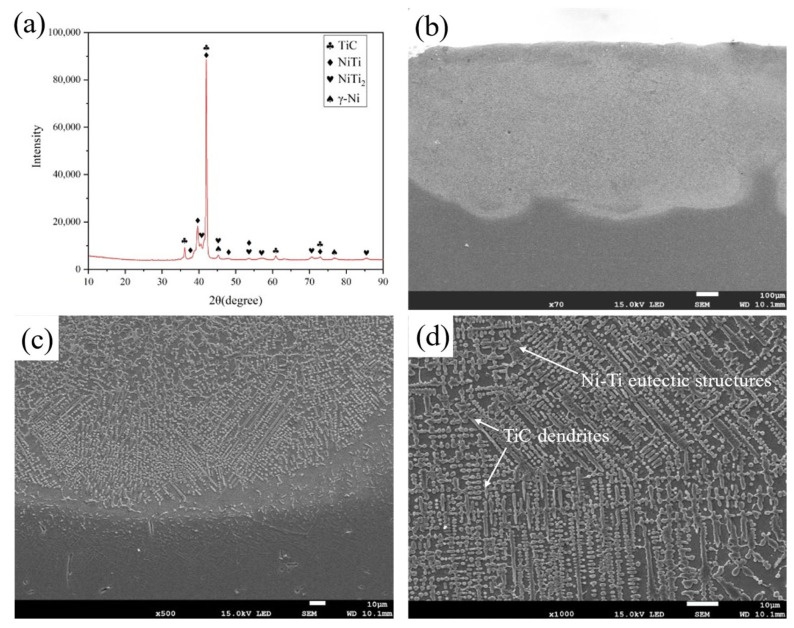
Phase compositions and microstructure morphology of the coating with nano-Nd_2_O_3_ addition: (**a**) XRD diffraction diagram; (**b**) Cross-section morphology; (**c**) Bonding zone morphology; (**d**) typical microstructure morphology.

Figure 2 shows the phase compositions and microstructure morphology of the coating with nano-Nd_2_O_3_ addition. The phases in the coating mainly include TiC, γ-Ni, NiTi and NiTi_2_ phases, differing only in the relative content with normal coating in our previous work, seen in Figure 2a [32]. With nano-Nd_2_O_3_ addition, the intensity of the TiC diffraction peak was improved, indicating that more TiC formed in the coating with nano-Nd_2_O_3_ addition. Nano-scaled rare earth oxides have the ability to reduce the surface tension and enhance the fluidity of the melt, which leads to a better wettability between the molten pool and the powders, and increases the diffusion efficient of Ti toward the carbon particles [33]. Thus, the quantity of TiC increased compared with coatings without the addition of nanoscaled rare earth oxides. 

Figure 2b shows the cross-sectional morphology of the coating at a low magnification. The size and density of the pores in the coating with nano-Nd_2_O_3_ addition are smaller than those without nano-Nd_2_O_3_ addition due to the improvement in the laser energy absorptivity [34], leading to an increased average melting depth. In the laser manufacturing process, laser energy absorptivity has an important effect on the quality of the forming coatings [35]. Due to the concentration of nano rare earth oxides, the total laser reflectivity is decreased, thus improving the laser absorptivity of the powder [34]. In addition, the surface-active rare earth oxide nano-Nd_2_O_3_ can increase the convective cycle strength in the molten pool [26,36]. Therefore, this process also helps the gas to escape from the molten pool more efficiently during the strong convective stirring process, reducing the formation of pores in the coating.

The morphology of the bonding zone is shown in Figure 2c. The coating has an excellent metallurgical bonding to the substrate without defects such as pores and cracks. Needle-like martensite formed in the bonding zone. Compared with the coating without nano-Nd_2_O_3_ addition, the needle-like martensite of the coating with 1.5 wt.% nano-Nd_2_O_3_ addition in Figure 2c is more refined, indicating that nanoscale rare earth oxide refines the structure efficiently. Rare earth elements are an outstanding surface modification addition due to their unique 4f electronic structure and strong metallicity, which has wide applicability for refining coating microstructures in laser alloying. Due to the larger atomic radius, rare earth elements in the solid solution could cause severe lattice distortion, increasing the system’s total energy. In order to keep the lowest free energy, rare earth atoms gather at grain boundaries and block the boundary migration, which would lead to the formation of nucleation sites, finally refining the microstructure [37]. The addition of rare earth oxides could also reduce surface tension and interface energy due to the surface activity, decreasing the critical nucleation work and contributing to homogeneous nucleation [38]. As a result, the solidification time is shortened and the dilution effect of the substrate is reduced. During the crystallization, the gathering of rare earth elements on the solid–liquid interface could reduce the diffusion and movement of the elements, which has decreased the dilution rate as well.

Figure 2d shows the typical microstructures of the coating, which is mainly composed of TiC dendrites with Ni-Ti eutectic structures [32]. TiC dendrites account for approximately 59.2% of the total microstructure, as measured by the area method. With the addition of nano-Nd_2_O_3_, the grains in the coating are refined, and TiC changes from coarse dendrites to fine dendrites, granular structures, and short rod-like structures that are closely arranged and evenly distributed. Compared with similar research [39,40], laser alloying G@Ni coatings with nano rare earth oxides addition have more refined TiC dendrites with an average width of about 1.4 μm.

### 3.2. High-Temperature Oxidation Resistance

**Figure 3:**
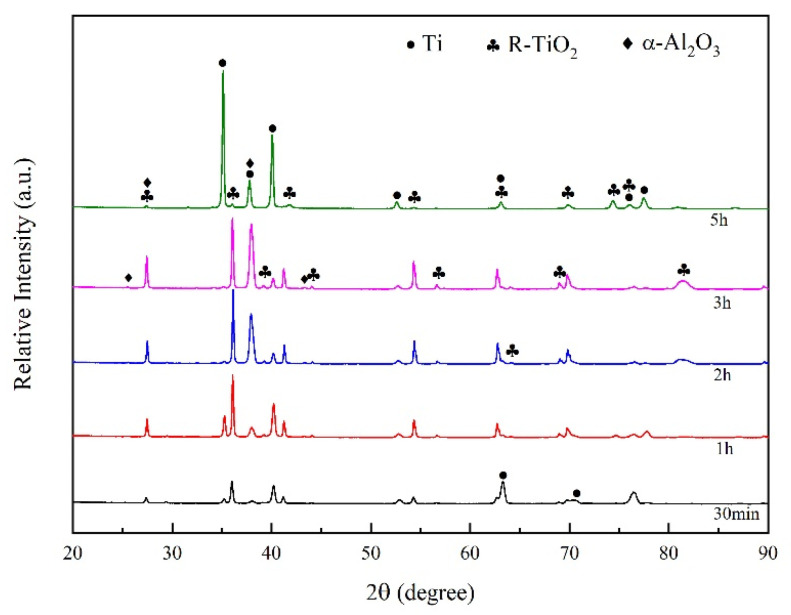
XRD Patterns of Ti-6Al-4V Alloy Oxidized at 800 °C.

The XRD results of the Ti-6Al-4V samples after 800 °C oxidation with different periods are shown in Figure 3. The oxide film grows cumulatively on the surface of the substrate. At the early stages of the oxidation, R-TiO_2_ forms by reaction between Ti and oxygen. Along the oxidation process, the oxide film gradually covers the whole surface, manifesting as the patterns of R-TiO_2_ gradually increase while the peaks corresponding to Ti decrease and finally disappear after 2 h of oxidation. A small amount of α-Al_2_O_3_ has formed after 3 h of oxidation. The final oxidation product of Ti-6Al-4V consists of R-TiO_2_ and α-Al_2_O_3_. After 5 h of oxidation, the peaks of Ti reappeared in the pattern, as the oxide film spalled while the substrate was exposed and further oxidized in the air.

**Figure 4:**
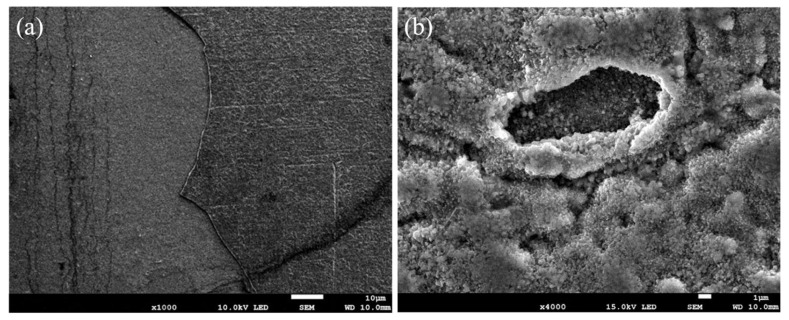
SEM morphologies of Ti-6Al-4V alloy oxidized at 800 °C for 5 h: (**a**) Macro morphology; (**b**) Micro morphology.

The surface morphology of the Ti-6Al-4V samples after 800 °C oxidation for 5 h is shown in Figure 4. Obvious cracks and pores appeared on the surface, and the inner alloy was exposed to the air and further oxidized, proving the poor high-temperature oxidation resistance of the Ti-6Al-4V alloy. The oxide film of the Ti-6Al-4V alloy mainly consists of R-TiO_2_, which grows via the in-diffusion of O and out-diffusion of Ti. At lower temperatures, most O elements form a solid solution with Ti, while only a small amount of O reacts with Ti to form a dense TiO_2_ oxide film. However, dense R-TiO_2_ oxide film would gradually crack at a higher temperature. O elements diffuse rapidly into the substrate, while Ti diffuses rapidly to the surface. As a result, the R-TiO_2_ oxide films cannot effectively protect the substrate from oxidation for the low bonding strength. Though α-Al_2_O_3_ has better stability at high temperatures, the ratio of R-TiO_2_ keeps increasing along with the oxidation, while the low content of Al elements limits the further formation of α-Al_2_O_3_.

**Figure 5:**
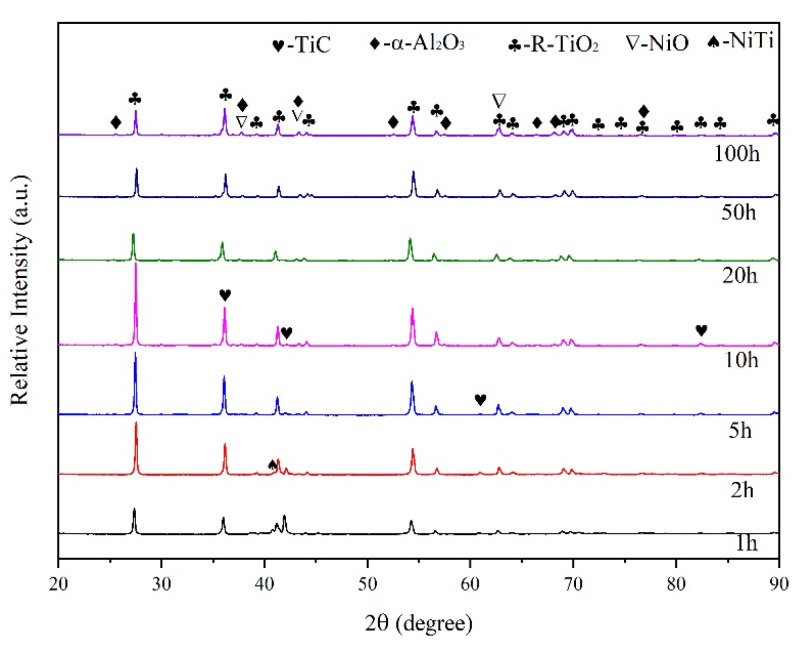
XRD pattern of the normal coating oxidized at 800 °C.

The XRD results of the normal coatings without nano-Nd_2_O_3_ addition after 800 °C oxidation with different durations are shown in Figure 5. The pattern of α-Al_2_O_3_ appeared after 2 h of oxidation. After 10 h, a small amount of NiO had formed. The final oxidation product of the coating consists of R-TiO_2_, α-Al_2_O_3_ and NiO, while the origin coating phase TiC and NiTi disappeared after 20 h of oxidation.

**Figure 6:**
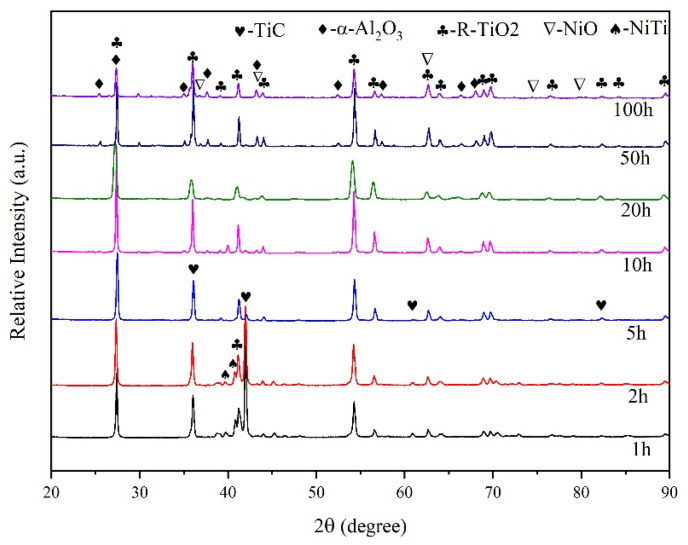
XRD pattern of the coating with nano-Nd_2_O_3_ addition oxidized at 800 °C.

The XRD results of the coatings with nano-Nd_2_O_3_ addition after 800 °C oxidation with different durations are shown in Figure 6. The oxidation products of the coatings with nano-Nd_2_O_3_ addition are similar to those without nano-Nd_2_O_3_, while they differ in appearance time and relative content. The origin coating phase TiC and NiTi disappeared after 10 h of oxidation since the rare earth addition aggregated on the coating surface and promoted the formation of the protective oxide film. The patterns of α-Al_2_O_3_ and NiO after 100 h of oxidation in Figure 6 are stronger than those in Figure 5, indicating that more α-Al_2_O_3_ and NiO were generated in coatings with nano-Nd_2_O_3_ addition.

**Figure 7:**
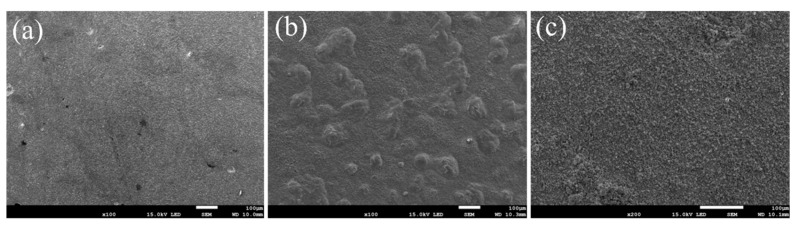
Surface morphologies of the normal coating oxidized at 800 °C: (**a**) 1 h, (**b**) 20 h, (**c**) 100 h.

Figure 7 shows the macro surface morphologies of the coatings oxidized at 800 °C within 1, 20, and 100 h. The oxides on the surface are hardly observed at the beginning (Figure 7a), while after 20 h, obvious granular oxides can be observed (Figure 7b). Finally, after 100 h of oxidation, the whole surface was covered by a dense and uniform oxide film, where no cracks, pores or spalling were observed (Figure 7c).

**Figure 8:**
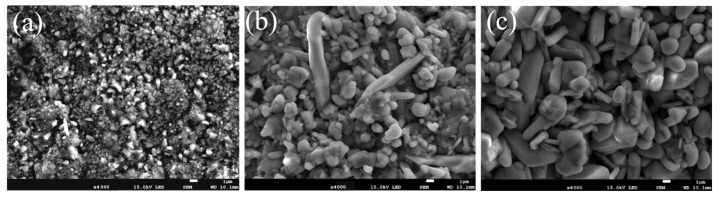
Micro surface morphologies of G@Ni laser alloying coatings oxidized at 800 °C: (**a**) 1 h, (**b**) 20 h, (**c**) 100 h.

**Figure 9:**
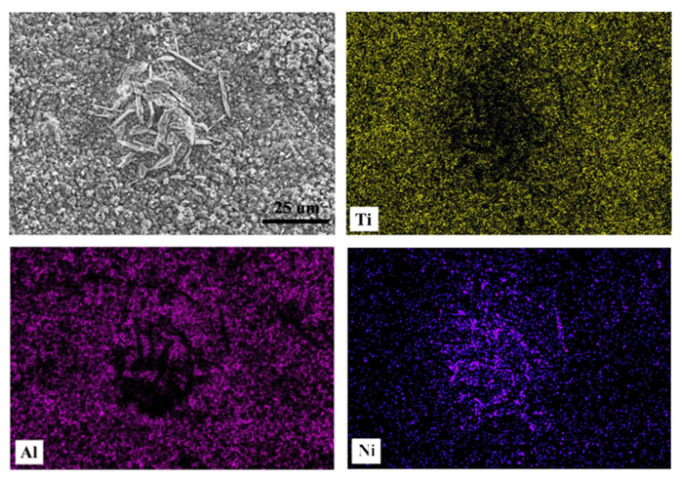
EDS results of the typical morphologies of the coating.

Figure 8 shows the morphologies of the coatings oxidized at 800 °C with 1, 20, and 100 h. At the beginning of the oxidation, granular oxides formed on the surface of the coating, then congregated as cellular structures with tiny granular oxides around. New tiny cellular oxides formed around the origin oxides as the oxidation process, and the size of the oxides kept growing until they transformed into granular oxides and covered the whole surface of the coating. After 20 h, the granular oxides grow up, where new tiny rod-shaped oxides are formed in between. Figure 9 shows the EDS results of the typical microstructure of the oxide films. The tiny granular oxides mainly consist of TiO_2_, while the cellular structures and the tiny rod-shaped oxides are Al_2_O_3_ and NiO, respectively.

The formation process of the oxide film can be concluded according to the microstructure changes of the oxide film. At the beginning of the oxidation, the O element was attached to the surface of the coating. Then, TiO_2_ and Al_2_O_3_ were nucleated and grew at positions with nucleation energy, which formed as cellular structures. With the oxidation process, the growth of cellular oxides was limited for the atom supply restriction, while the new tiny cellular oxides still presented high growth speeds. NiO subsequently formed between the TiO_2_ and Al_2_O_3_. Finally, the oxides congregated and covered the whole surface of the coating. The protective effects of the oxide film are attributed to the fact that NiO has a double-layer structure, where the out layer is denser with tensile stress. During the oxidation and cooling process, the tensile stress is offset by thermal stress. Finally, the NiO oxide film sustains only a small pressure stress, protecting the bonding between the oxide film and the substrate.

**Figure 10:**
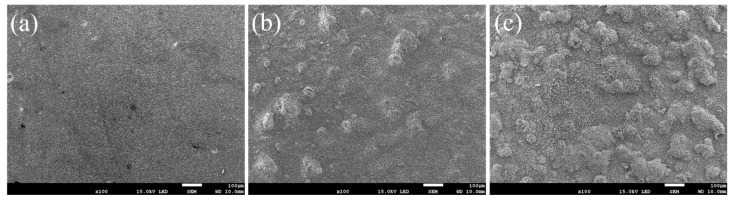
Macro surface morphologies of coatings with nano-Nd_2_O_3_ addition oxidized at 800 °C: (**a**) 1 h; (**b**) 20 h; (**c**) 100 h.

**Figure 11:**
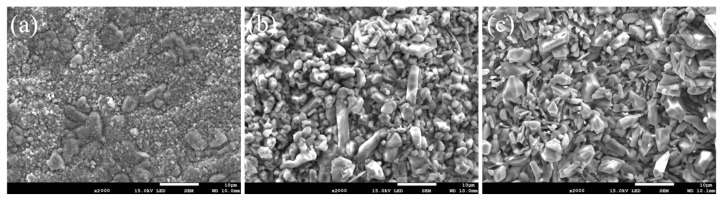
Surface morphologies of coatings with nano-Nd_2_O_3_ addition oxidized at 800 °C: (**a**) 1 h; (**b**) 20 h; (**c**) 100 h.

Figure 10 and Figure 11 show the morphologies of the coating with the addition of 1.5 wt.% nano-Nd_2_O_3_ oxidized at 800 °C for 1, 20, and 100 h. The microstructure of the oxide film is finer and denser than that of normal coatings, without any spalling. Compared with oxide film without rare earth addition, the density of tiny rod-shaped NiO is higher since the rare earth element Nd could promote the out-diffusion of Ni, thus increasing the oxidation velocity of Ni. With a larger content of protective NiO, the oxide film on the coating with nano-Nd_2_O_3_ addition has more effective protection on the substrate. With the microstructure refining effect of nano-Nd_2_O_3_ addition, the fine grains of the oxide film possess a good plasticity, which would improve the spalling resistance of the film [41].

**Figure 12:**
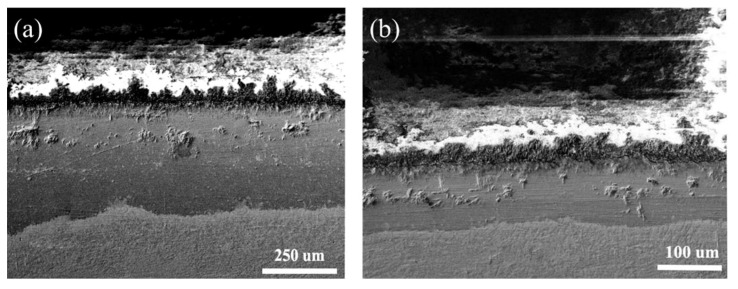
Cross-section morphology of the coatings oxidized at 800 °C for 100 h: (**a**) The normal G@Ni coating, (**b**) the coating with nano-Nd_2_O_3_ addition.

Figure 12 shows the cross-section morphology of the normal G@Ni coating and the coating with nano-Nd_2_O_3_ addition oxidized at 800 °C for 100 h. The thickness of the oxide film of the normal coating is about 300 μm, while that of the coating with nano-Nd_2_O_3_ addition is about 100 μm. The rare earth oxides affect the mass transmission of elements during oxidation by segregation among grain boundaries, which improves the high-temperature oxidation resistance. During the oxidation process, a large number of metal cations diffuse outward, leaving vacancies in the loose oxide film. With rare earth oxide addition, oxidative segregation would cause lattice distortion at grain boundaries, which hinders the outward diffusion of metal cations. The oxidation process relies on the diffusion of O^2−^, which slows the velocity of oxidation, thus reducing the thickness of the oxide film. It is noticeable that the thickness of the oxide film distributes unevenly since oxidation prefers to happen at defects like pores or cracks rather than in flat and dense regions.

**Figure 13:**
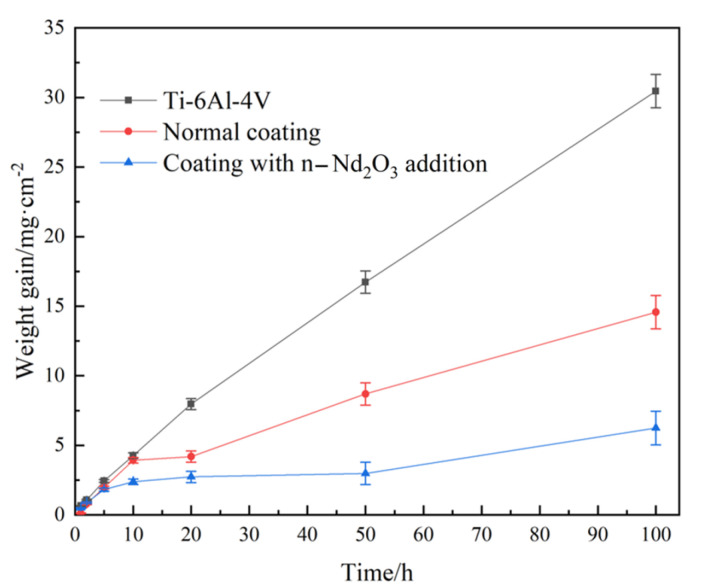
Isothermal oxidation kinetic curve of the Ti-6Al-4V alloy and the coatings.

To further characterize the high-temperature oxidation resistance of the coatings, we weighed the oxidation weight gain per unit of the samples after 1, 2, 5, 10, 20, 50, and 100 h of 800 °C oxidation. Figure 13 shows the isothermal oxidation kinetic curve at 800 °C of the Ti-6Al-4V substrate and the coating with and without rare earth oxides. The weight gain of Ti-6Al-4V after 100 h of oxidation is 30.458 mg/cm^2^, and the linear fitting curve proves the poor protection of the oxide film. Meanwhile, the oxidation weight gain of the coating without rare earth oxides after 100 h of oxidation is 14.571 mg/cm^2^, which is about 1/2 of the substrate. The parabola curve also proves the good protection effect. The weight gain speed is higher at the beginning while decreasing as the oxidation progresses due to the difference between oxidation factors in different oxidation stages. The oxidation weight gain of the coating with nano-Nd_2_O_3_ addition after 100 h of oxidation is 6.244 mg/cm^2^, which is only 1/5 of the substrate, proving that the rare earth oxide addition could practically decrease the oxidation speed of the coating.

Overall, the improvement of the high-temperature oxidation resistance of the nanoscaled Nd_2_O_3_ addition in the G@Ni laser alloying coating has been proved by this study. However, the effect of the addition amount of nano-Nd_2_O_3_ has not yet been studied. In the future, we will further investigate the effect of the addition amount of nanoscaled rare earth oxides on the high-temperature oxidation resistance of the laser alloying G@Ni coatings on titanium alloy. The effect of other types of nanoscaled rare earth oxides, such as n-La_2_O_3_ and n-Y_2_O_3_, on the high-temperature oxidation resistance of the laser alloying G@Ni coatings on titanium alloy is also a future direction of study. Meanwhile, the effects of nanoscaled rare earth oxides addition on the high-temperature properties of laser alloying coatings, such as the high-temperature corrosion resistance and high-temperature fatigue properties of the coatings, also need to be further investigated. Computational simulation is an effective, economical and convenient analytical method in material science, especially in the titanium alloy field [42,43]. We would combine computational simulation and experimental testing for further high-temperature properties studies of the laser alloying coatings on titanium alloys in the future.

## 4. Conclusions

This study focused on the microstructure and high-temperature oxidation properties of laser alloyed coatings on Ti-6Al-4V with and without the addition of rare earth oxidation nano-Nd_2_O_3_. With the same phase content of TiC, NiTi, NiTi_2_ and γ-Ni, the microstructure of the coating with nano-Nd_2_O_3_ addition is finer than that of the normal coating due to the microstructure refinement and dilution-reducing effect of nano-Nd_2_O_3_. After 100 h of 800 °C heating, oxide films consisting of R-TiO_2_, α-Al_2_O_3_ and NiO formed on the surface of the laser alloying coatings without obvious spalling. Meanwhile, the oxide films on the surface of the Ti-6Al-4V substrate start spalling under 800 °C after only 2 h. With nano rare earth oxidation addition, more NiO formed in the oxide film, which effectively strengthens the protective effect of the film. After 100 h of 800 °C oxidation, the depth of the oxide film decreases due to the improvement of oxidation properties, and the oxidation weight gain of the normal coating is 14.571 mg/cm^2^, while that of the coating with nano-Nd_2_O_3_ addition is 6.244 mg/cm^2^, further proving that the nano-Nd_2_O_3_ addition substantially improved the high-temperature oxidation properties of the coating. In the future, this study will further investigate the effect of the type and addition amount of nanoscaled rare earth oxides addition on the high-temperature properties of laser alloying coatings using computational simulation and experimental testing.

## Data Availability

The data presented in this study are available on re-quest from the corresponding author.
